# Preventing white spot lesions around orthodontic brackets: efficacy of pre-reacted glass-ionomer barrier coat versus silver diamine fluoride: an in vitro study

**DOI:** 10.1038/s41405-025-00332-w

**Published:** 2025-05-05

**Authors:** Enas A. Elshenawy, Safa B. Alawy, Wafaa Yahia Alghonemy, Ahmed Ibrahime El dosoky

**Affiliations:** 1 Dental Biomaterials Department, Faculty of Dentistry, Tanta University, 31773 Tanta, Egypt; 2 Department of Orthodontics, Faculty of Dentistry, Tanta University, 31773 Tanta, Egypt; 3 Associate Professor at Basic Dental Sciences Department, Faculty of Dentistry, Zarqa University, 13110 Zarqa, Jordan; 4 Associate Professor at Oral Biology Department, Faculty of Dentistry, Tanta University, 31773 Tanta, Egypt; 5 Pediatric, Oral Health and Preventive Department, Faculty of Dentistry, Tanta University, 31773 Tanta, Egypt

**Keywords:** Preventive dentistry, Dental materials

## Abstract

**Objectives:**

to compare the effect of using a pre-reacted glass-ionomer (PRG) barrier coat versus silver diamine fluoride (SDF) varnish treatment before orthodontic bracket bonding to prevent white spot lesions (WSL). The effect of these materials on the shear bond strength of orthodontic brackets was evaluated through an in vitro study.

**Methods:**

One hundred-five premolars were used; forty-two specimens were designated for comparing SDF versus PRG-barrier coat using a scanning electron microscope (SEM) with elemental analysis and microhardness testing (*n* = 21/group). Tested materials were applied in a split-tooth design protocol (side A: control, side B: treated). Measurement was made at baseline and after bracket placement and exposure to the pH cycle. A polarized light microscope (PLM) was used for qualitative examination. Sixty-three specimens were intended for shear bond strength (SBS) testing after the pH cycle as follows: control group (no treatment), SDF group, and PRG-barrier coat group (*n* = 21/group). Statistical analysis was done using Paired T-test.

**Results:**

SEM for specimens treated by SDF or PRG revealed enamel remineralization with calcium deposits and small porosities between the crystallites. All groups had a significant difference regarding the Ca/P ratio and microhardness. Baseline hardness for both groups was significantly higher than the treated sides, which was significantly higher than the non-treated sides (*P *= 0.000*) and (*P *= 0.000*) in accordance with the Paired T-test. In comparing SDF with PRG-coat groups, the T-test showed non-significant differences in mean differences between treated and baseline hardness values (*T *= 0.32, *P *= 0.74). PLM for treated specimens by SDF or PRG depicted an evident remineralized surface enamel layer. SBS values did not differ significantly between groups.

**Conclusions:**

As confirmed by SEM and PLM, applying either SDF varnish or PRG-barrier coat before bonding orthodontic brackets could effectively prevent the development of WSL and achieve surface enamel protection. In addition, the two applied varnishes showed slightly higher shear bond strength of orthodontic brackets compared to the control group, with the SDF slightly higher than PRG. Also, clinical translation is needed in future research to evaluate the study.

## Introduction

Enamel demineralization, presented clinically as a white spot lesion near orthodontic bands and brackets, continues to be a clinical concern. Multiple investigators have documented the occurrences of these “white spot lesions” as early as one month after initiating orthodontic treatment [1–6].

Orthodontists have long endeavored, with limited success, to decrease demineralization. The preventive effects of dentifrices and/or home use of fluoride solutions, for example, have been established; nevertheless, patient compliance with the traditional preventive measures is also an issue. It was shown that 52.5% of the patients did not utilize fluoride solutions at home [1, 7, 8].

It would be more logical to prioritize preventive methods that do not rely on patient compliance for the usual orthodontic patient population. These, namely adolescents, are already more prone to tooth caries [9]. Remineralizing therapy has recently gained popularity, and many studies suggest that they are as effective as traditional restorative approaches [10–13].

Fluoride application was the gold standard for preventing the episode of demineralization. However, after applying fluoride varnish, the outer layer may become saturated with more minerals, which reduces ion diffusion to the deepest layer [14]. Several remineralizing agents were employed to deposit calcium/phosphate minerals in enamel that resemble hydroxyapatite; nevertheless, the crystalline deposition of minerals does not mimic the natural one [15]. Moreover, other materials used in preventing WSL as chlorhexidine, nano hydroxy appetite, self-assembling peptide p11-4, and casein phosphopeptide-amorphous calcium phosphate [16].

Bioactive substances with the power of remineralization at the deep area of the body of the lesion were utilized. Silver Diamine Fluoride (SDF) is a pharmaceutical bioactive agent that arrests caries [17]. Its mechanism of action is predicated upon its capacity to enhance the microhardness of enamel surfaces and mitigate mineral loss [18]. Over 20 clinical investigations completed globally have provided evidence of the efficacy of SDF in halting the progression of dental caries [19]. A recent systematic analysis examined the efficacy of 38% SDF in halting primary teeth lesions. The findings revealed an overall success rate of 81%, indicating the potential of this approach as a promising strategy for preventing white spot lesions around orthodontic brackets [20]. However, the main disadvantage of SDF is the black discoloration of teeth due to silver phosphate formation; thus, it is contraindicated in anterior teeth [21].

Another bioactive option is surface reaction-type pre-reacted glass ionomer (S-PRG) fillers containing dental materials. PRG filler was an effective additive due to its capacity to liberate and replenish fluoride ions [22]. Further, it releases additional active ions when exposed to water or acidic solutions. These ions can modulate acidic environments, turning the surrounding environment into reduced coating material (PRG Barrier) to reduce dentin hypersensitivity and prevent cavities on smooth surface areas [23, 24].

This material incorporates S-PRG filler, which releases fluoride. Two previous studies have examined the effect of PRG coating material as a preventive method, and it was found to have the ability to prevent enamel demineralization and microbial adhesion [24, 25].

To our knowledge, no previously published study evaluated the capacity of remineralizing materials to prevent white spot lesions around orthodontic brackets before bonding them and evaluating them in conditions that include easy food retention and a high acidic challenge. So, the purpose of this study was to compare the preventive potential of silver diamine fluoride versus PRG barrier coat on the development of White spot lesions (WSLs) around orthodontic brackets regarding surface elemental analysis and microhardness. In addition, the effect of these materials on the shear bond strength of orthodontic brackets was evaluated. The first null hypothesis states that surface elemental analysis and microhardness will not change significantly after tested materials are applied. The second null hypothesis states that the applied, tested materials will not interfere with the SBS of orthodontic brackets to the enamel.

## Materials and methods

### Materials

The materials that were used in this study are presented in Table (1).

**Table 1:** Detailed description of composition of all materials used in the study.

Material	Composition	Manufacturer
Artificial saliva	(Na-3PO4 (3.90 mM), NaCl (4.29 mM), KCI (17.98 mM), CaCl2 (1.10 mM), MgCl2 (0.08 mM), H2SO4 (0.50 mM), NaHCO3 (3.27 mM)4	Laboratory prepared in Department of Chemistry –Faculty of Science.
38% SDF solution	Silver particles and 38% (44,800 ppm) fluoride ion, which at pH 10 is 25% silver, 8% ammonia, 5% fluoride, and 62% water.	Tooth Mate Company, Egypt
PRG Barrier Coat	**Base:** S-PRG filler based on fluoroboroaluminosilicate glass, Distilled water, Methacrylic acid monomer, and others **Active:** Phosphonic acid monomer, Methacrylic acid monomer, Bis-MPEPP, Carboxylic acid monomer, TEGDMA, Polymerization initiator, and others	Shofu. Dental Corp., Kyoto, Japan
Ortho Solo Primer	Highly filled light-cure adhesive, Bis-GMA resin	Ormco Corporation, Glendora, CA, USA
Grengloo (Two-way color change adhesive)	Uncured methacrylate ester monomers (20–38%), inert mineral fillers, fumed silica, activators, and preservative	Ormco Corporation, Glendora, CA, USA
Demineralizing solution	50 mMol acetic acid derivation, 2.25 mMol CaCl2 2H2O, 1.35 mMKH2PO4; 130 mm KCl 4	Laboratory prepared in Department of Chemistry –Faculty of Science
Remineralizing solution	(1.5 mMol Calcium Chloride-0.9 mMol Sodium Phosphate-150 mMol Potassium Chloride)	Laboratory prepared in Department of Chemistry –Faculty of Science.

### Study design and sample size calculation

This study was conducted as an in vitro experimental study using a split-tooth design, designed by the guidelines of a research ethics committee.

The specimens were designed to be surface characterized twice (before bonding and after bracket removal) by SEM, EDX, and microhardness. In addition, the effect of the tested materials on brackets’ shear bond strength was studied.

GPower, version 3.1.9.2, was used to determine the necessary sample size [26]. Based on the findings of an earlier study [10], the outcomes were reported as 45.9 ± 11.12 for giomer, 36.10 ± 9.95 for SDF and 34.0 ± 8.37 for control group. The effect size was calculated automatically, with the value being 0.513. The sample size was estimated, adopting a power of 95% and a significant level of 5%. The estimated sample size of 21 samples per group was found to be the bare minimum needed.

105 extracted human permanent maxillary first premolars were chosen as the sample based on the specified inclusion and exclusion criteria. Teeth were examined under a stereomicroscope (SZ-Olympus, Japan) using 10x magnification. Sound completely formed maxillary first premolars typically removed for orthodontic treatment were the inclusion criterion. The following teeth met the exclusion criteria: those with evident buccal flaws, microcracks, erosions, caries, or restorations [27].

A pilot study was conducted prior to the main experiment to refine the methodology, assess feasibility, and ensure consistency in data collection.

### Sample preparation

**Fig. 1:**
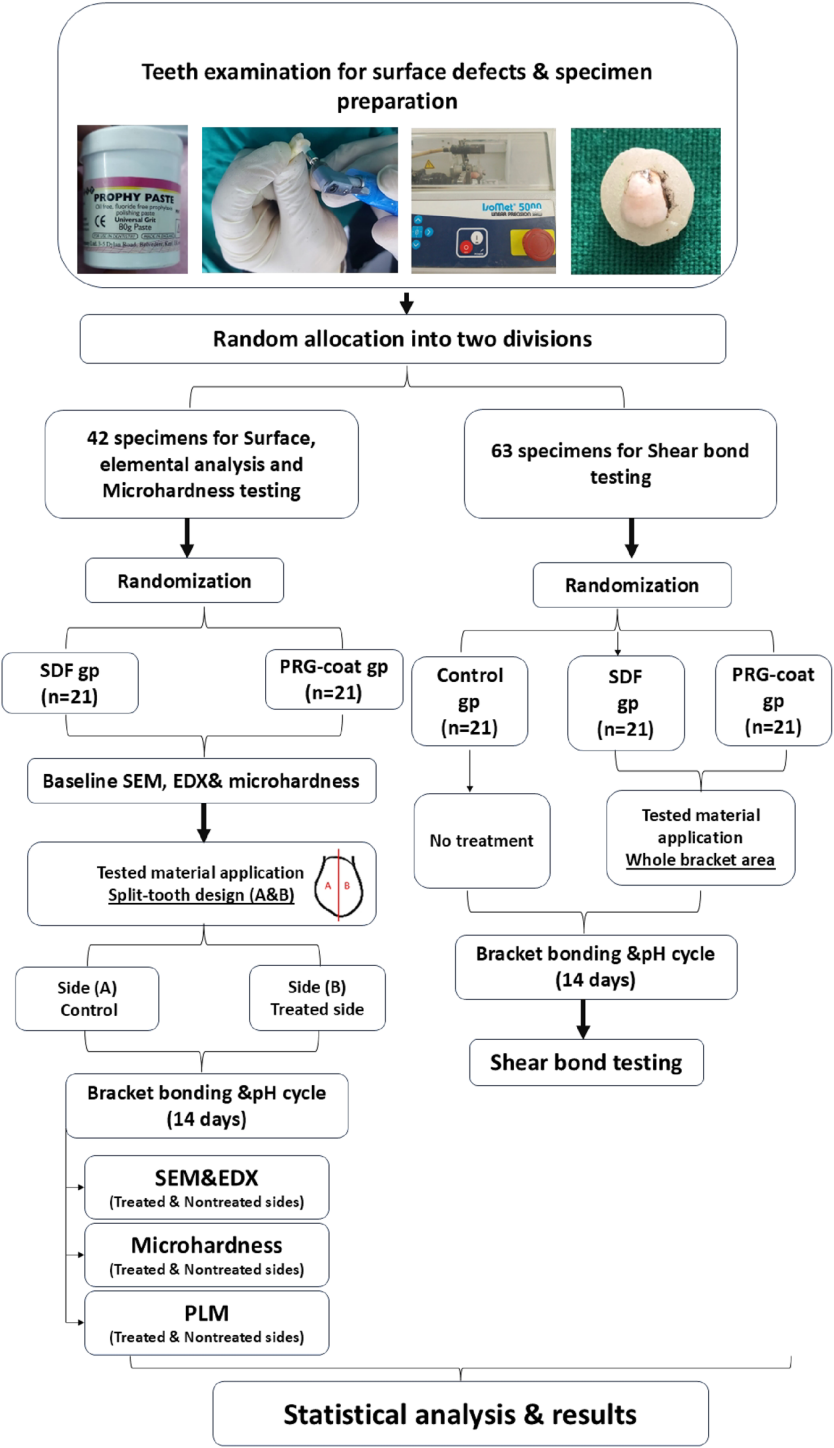
Flow chart explaining the workflow of the study.

After extraction, a hand scaler (Scaler 10 A, NOVA Instruments Ltd, UK) was used to remove calculus and soft deposits from the teeth. The teeth were carefully washed with distilled water after being cleaned with fluoride- and oil-free pumice (Prophy paste, PSP Dental Company Ltd, UK). Teeth crowns were separated at CEJ using a diamond saw (IsoMet precision saw, Buehler, UK). Each tooth’s coronal portion was subsequently embedded in self-cured acrylic resin blocks (Acrostone, Anglo Egyptian Company, Egypt) with buccal surfaces facing upward. The teeth were housed in laboratory-prepared artificial saliva in an incubator at 37 ° C, which was changed daily until the experiment was finalized (Fig. 1).

### Sample assignment

For elemental analysis and microhardness, the buccal surfaces of 21 specimens from each group were divided vertically into two halves by a permanent marker. The control side (section A) received no treatment in these specimens. The other half was the test side, where the enamel surface would be treated with its respective remineralizing anti-cariogenic agent (section B) (Fig. 1).

For shear bond testing, three groups (*n* = 21/group) were allocated randomly: the Control group, which received no treatment before bracket bonding; the SDF group, which received SDF varnish on the whole bracket area; and the PRG-coat group, which received a PRG-barrier coat over the entire bracket area.

### Application of tested material

#### SDF application

The buccal surface was etched with 37% phosphoric acid etching gel for 30 s, followed by a water rinse for 30 s and air dried gently. After that, a micro brush was used to apply a single coat of 38% SDF solution (Toothmate company, Egypt) directly to the tooth surface [28]. To ensure SDF’s penetration, it was left on for 3 min, then excess SDF was removed with a cotton pellet, and then the buccal surface was water rinsed for 30 seconds and air-dried for 5 seconds.

#### PRG coat application

After acid etching the enamel surface as described for the previous group, the base and Active components of the PRG coat (Shofu. Dental Corp., San Marcos, CA, USA) were mixed using a disposable brush. Then, a thin layer of the mixture was applied to the dried tooth surface, left undisturbed for more than 3 sec, and light-cured using a dental light-curing unit for 10 sec.

### Bracket placement

After the tested material application, a uniform thin layer of liquid primer Ortho Solo™ (primer, Ormco, USA) was applied, air blown to dry into a thin film. The metal bracket (premolar brackets, MBT 0.022′′, American orthodontics, /base surface area of 10.25mm^2^) was coated with an adhesive (Grengloo^TM^, two-color change adhesive, Ormco, USA), then brackets were placed on the buccal surface parallel to the tooth’s long axis, in the middle of the occluso-gingival joint, and at the height of the contour mesiodistally. Afterward, the composite was light-cured for 40 seconds.

### pH cycling

All groups’ samples underwent remineralization/demineralization pH cycles for 14 days. Each cycle consisted of four phases: a demineralizing phase lasting 120 minutes, a washing phase lasting 30 seconds, a remineralizing phase lasting 60 minutes, and a final washing for 30 seconds. The specimens were subjected to the remineralizing solution for a 6-hour “night” period [27]. Table 1 lists the ingredients of the demineralizing and remineralizing solutions [29]. The pH of both solutions was measured using a pH meter.

### Surface characterization

Specimens designated for surface evaluation by SEM, microhardness, and PLM were assessed as follows:

#### Scanning electron microscopy (SEM) and quantitative elemental analysis (weight %) by EDX spectrometry

Sample surfaces on sections A and B were examined using SEM attached with EDX Unit SEM (JEOL-JSM-5200LV, Tokyo, Japan). **For SEM evaluation**, the samples were carefully dried and gold-plated. Then, the samples were fixed to investigate the enamel surface. In addition, Both Calcium (Ca) and Phosphorus (P) content at the enamel surface of each specimen were analyzed quantitively as weight percentage using EDX. This step was done for all specimens at baseline and after bracket removal for non-treated (section A) and treated sides (section B).

#### Microhardness

Microhardness was measured using a Digital Vickers Microhardness testing machine (ZwicRoell, West Midlands, England) using 300 gm force for 10 seconds with a Vickers’ diamond indenter and 10X objective length. Three indentations were made on the surface of each specimen, and the average was calculated. This step was done for all specimens at line baseline and after bracket removal (for treated and non-treated sides).

#### Polarized light microscopic examination (PLM)

Each tooth was sectioned vertically in a buccolingual direction utilizing a diamond saw (IsoMet precision saw, Buehler, UK) to split up section A from section B. Each half could be analyzed individually under PLM (Olympus America Inc.). According to Yadav et al. [30], a manual grinding procedure was employed to obtain a buccolingual slice of each specimen (150 μm-thick). Those sections were washed under running water, cleaned in xylene for one minute, and mounted on microscopic slides. Then, the slides were examined under PLM to assess the test area qualitatively. The images were captured using a PLM built-in camera (LEICA ICC50 HD Camera system) via image software LAS EZ version 3.0.0.

### Shear bond strength (SBS) evaluation

**Fig. 2:**
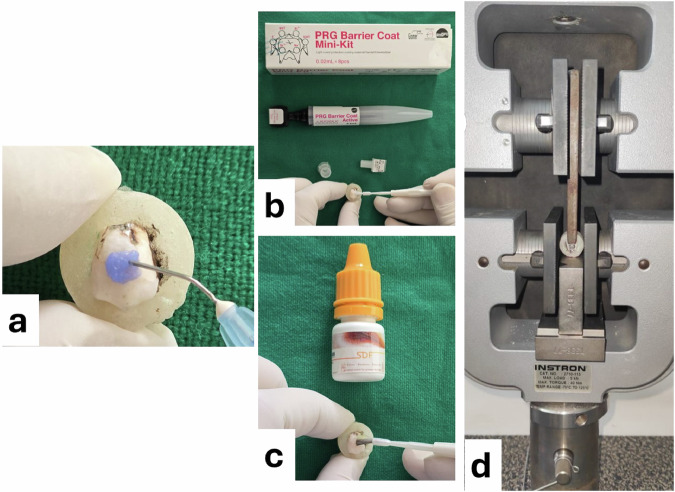
Shear bond strength (SBS) evaluation. **a** Phosphoric acid etching, **b** PRG-coat application, **c** SDF application, **d** shear test using universal testing machine.

Specimens designated for SBS evaluation were examined: A universal testing machine (INSTRON 3365, USA) was used to measure SBS. A chisel was applied at 1 mm/min cross‑head speed at the bracket–tooth interface until debonding. The debonding force was measured in Newtons (N) and then divided by bracket base area (10.25 mm^2^) to calculate SBS in MPa. (Fig. 2).

### Examiner reliability

To ensure consistency in data collection, each examiner independently assessed the same samples under standardized conditions. The degree of agreement between examiners was evaluated using Cohen’s kappa (κ) test, yielding a kappa value of 0.9, which reflects excellent agreement.

### Statistical analysis

The raw data of the study was tested for their normal distribution using Shapiro-Wilk test. Normality was assured numerically (Sig.>0.05). The quantitative data of Ca/P ratio and VHN, being measured repeatedly at the same specimen at different conditions, were statistically analyzed using a Paired T-test between each two conditions. The mean difference between the baseline and treated side for each group was calculated for the Ca/P ratio and VHN, followed by a T-test to compare the effectiveness of each material in preserving baseline tooth condition. For SBS (MPa), statistical analysis was done using one-way analysis of variance (ANOVA). The analysis was performed by IBM SPSS software package version 20.0. **(**Armonk, NY: IBM Corp**)**.

## Results

### SEM & EDX spectrometry results

**Fig. 3:**
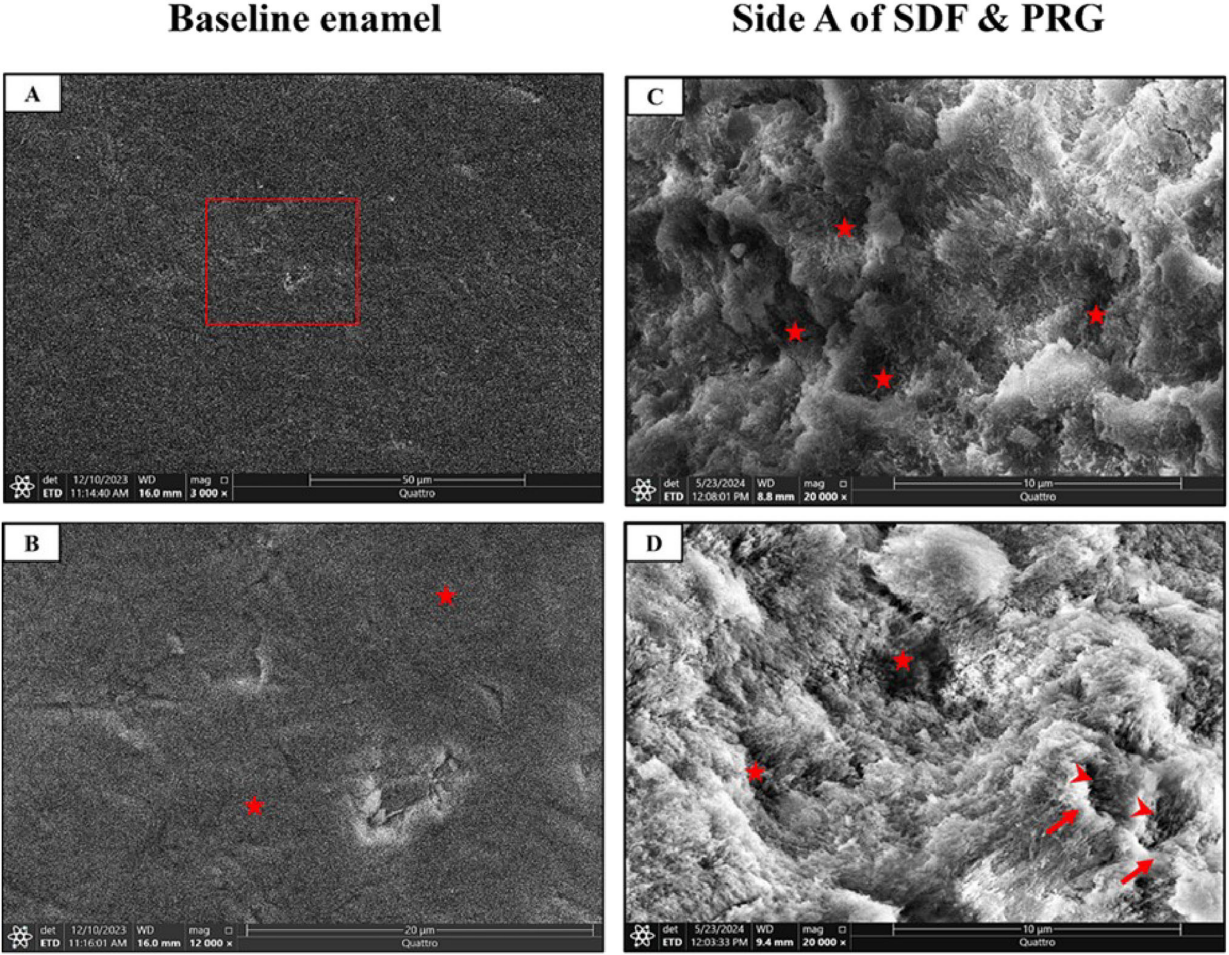
Scanning electron micrographs of typical enamel surface (Baseline) and side A (untreated side) of SDF and PRG groups. **A** Normal enamel surface (Baseline) showing smooth enamel architecture. **B** A higher magnification of the red boxed area at A shows smooth enamel with a prismatic enamel (stars) covering its external surface. **C** The typical etching pattern in all groups and the irregular etching pattern with uneven depressions (stars). **D** Combination between irregular depressions pattern of etching (stars) and type-I pattern, which includes removal of the rod body (arrowheads) and maintenance of rod boundary and interrod area (arrows). (SEM, original magnification; **A** x 3000, **B** x 12.000, **C** &**D** x 20,000).

**Fig. 4:**
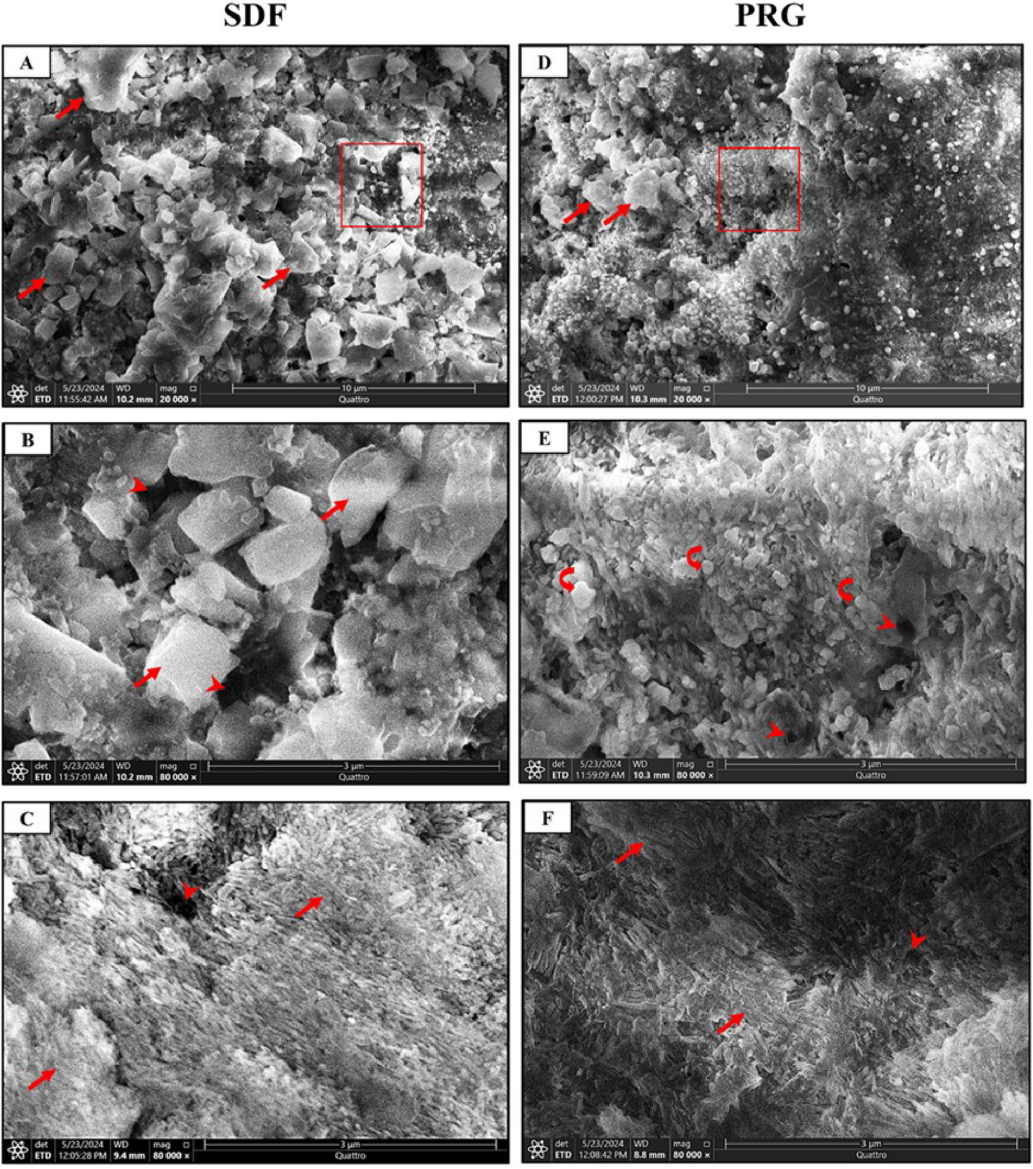
Scanning electron micrographs of side B (treated side) of SDF and PRG groups. **A** **SDF-B** showing enamel remineralization covers nearly the whole enamel surface with many large calcium crystals (arrows). **B** A higher magnification of the red boxed area at A shows tiny pores (arrowheads) between the calcium crystallites (arrows). **C** **SDF-B** showing complete enamel remineralization with coalescence of the calcium crystallites (arrows) and surface micropores (arrowheads). **D** **PRG-B** showing enamel remineralization covers nearly the enamel surface with many large calcium deposits (arrows). **E** A higher magnification of the boxed area at D shows small porosities (arrowheads) between the calcium crystallites and residuals of PRG particles (curved arrows) adhering to the enamel surface. **F** **PRG-B** showing complete enamel remineralization with coalescence of the calcium crystallite (arrows) and surface micropores (arrowheads). (SEM, original magnification; **A**, **D** x 20.000, **B**, **E**, **C** & **F** x 80.000).

To better understand the alterations occurring after treatment application, we captured an SEM image of a typical, intact enamel surface (Baseline). The normal enamel surface appeared smooth in architecture with a layer of a prismatic enamel covering its external surface (Fig. 3A, B). Side A of both SDF and PRG groups (untreated side) depicted the typical etching pattern irregular with uneven depressions and type-I pattern which include removal of the rod body and maintenance of rod boundary and interrod area (Fig. 3C, D). However, side B of SDF (treated side) showed enamel remineralization that covered almost the whole enamel surface with many large calcium crystals combined. Tiny pores between the calcium crystallites were also depicted in small areas (Fig. 4A–C). Moreover, side B of PRG (treated side) revealed enamel remineralization, which covered almost the enamel surface with many large calcium deposits coalesced with small porosities between the calcium crystallites (Fig. 4D–F).

The data obtained from EDX spectrometry was Ca and P weight %, so we calculated Ca/P ratios for each group based on their content (Table 2). Paired T-test results of both groups showed significant differences between Ca/P ratios at baseline and non-treated sides (*P *= 0.000*). In addition, the treated side of both groups showed significantly higher Ca/P ratios than those of non-treated sides (*P *= 0.000*). Regarding the SDF group, the Ca/P ratio of the treated side did not differ significantly from the baseline ratio (*T *= 1.6, *P *= 0.121). For the PRG-coat group, the Ca/P ratio of the treated side showed a significantly lower Ca/P ratio than baseline ratios (*T *= 3.4, *P *= 0.003*). Notably, non-significant differences were found in the mean difference of Ca/P ratios between treated and baseline groups according to T-test outcomes (*T *= 0.99, *P *= 0.32) (Table 3).

**Table 2:** Mean values, standard deviation, and statistical analysis for Ca/P ratios and VHN of all groups.

(A) Mean values of (Ca/P) ratio in all groups				
			Mean ± SD	Min-Max
Ca/P Ratio	**SDF**	**Baseline**	2.08 ± 0.11	1.83- 2.43
		**SDF-A**	1.04 ± 0.09	0.87- 1.19
		**SDF-B**	2.00 ± 0.17	1.78- 2.47
	**PRG-coat**	**Baseline**	2.06 ± 0.15	1.67- 2.45
		**PRG-A**	1.05 ± 0.14	0.83- 1.39
		**PRG-B**	1.9 ± 0.11	1.67- 2.11
VHN	**SDF**	**Baseline**	421.44 ± 26.45	377- 475
		**SDF-A**	237.99 ± 36.63	172- 303
		**SDF-B**	334.23 ± 31.28	289- 400
	**PRG-coat**	**Baseline**	428.95 ± 33.87	364- 488
		**PRG-A**	235.37 ± 28.85	187-300
		**PRG-B**	344.81 ± 25.66	306- 387

**(B) Paired T-test results for Ca/P ratio and VHN**				
			**T**	***P***-**Value**
Ca/P Ratio	**SDF**	**Baseline& SDF-A**	28.2	0.000*
		**Baseline& SDF-B**	1.6	0.121
		**SDF-A& SDF-B**	21.8	0.000*
	**PRG-coat**	**Baseline& PRG-A**	18.8	0.000*
		**Baseline& PRG-B**	3.4	0.003*
		**PRG-A & PRG-B**	18.4	0.000*
VHN	**SDF**	**Baseline& SDF-A**	19.7	0.000*
		**Baseline& SDF-B**	11.2	0.000*
		**SDF-A& SDF-B**	12.2	0.000*
	**PRG-coat**	**Baseline& PRG-A**	25.2	0.000*
		**Baseline& PRG-B**	15.2	0.000*
		**PRG-A & PRG-B**	17.8	0.000*

* Indicated significance.

**SDF-A**: Untreated side of SDF gp, **SDF-B**: Treated side of SDF gp, **PRG-A**: Untreated side of PRG gp, **PRG-B**: Treated side of PRG gp.

**Table 3:** T-test comparing the mean difference between baseline and treated side for each group.

		Mean difference	T	*P*-Value
Ca/P Ratio	**SDF**	0.088	0.99	0.32
	**PRG-coat**	0.16		
VHN	**SDF**	87.2	0.32	0.74
	**PRG-coat**	84.1		

### Microhardness results

All groups’ mean VHN and standard deviations are presented in (Table 2). Baseline hardness for both groups was significantly higher than the treated sides, which was significantly higher than the non-treated sides (*P *= 0.000*) and (*P *= 0.000*) in accordance with the Paired T-test. In comparing SDF with PRG-coat groups, the T-test showed non-significant differences in mean differences between treated and baseline values of VHN (*T *= 0.32, *P *= 0.74) (Table 3).

### Shear bond strength results

Mean SBS values and standard deviations for all groups are presented in Table 4. One-way ANOVA showed a non-significant difference between the groups (*F *= 2.51, *P *= 0.089), with the SDF group showing slightly higher values followed by PRG-coat groups than the control group.

**Table 4:** Mean values, standard deviation, and statistical analysis for SBS of all groups.

Mean values of SBS in all groups and ANOVA results				
	Group	Mean ± SD	*F*	*P* Value
SBS	**Control**	10.77 ± 1.01	2.51	0.089
	**SDF**	11.6 ± 1.13		
	**PRG-coat**	10.97 ± 1.57		

### Polarized light microscope results

**Fig. 5:**
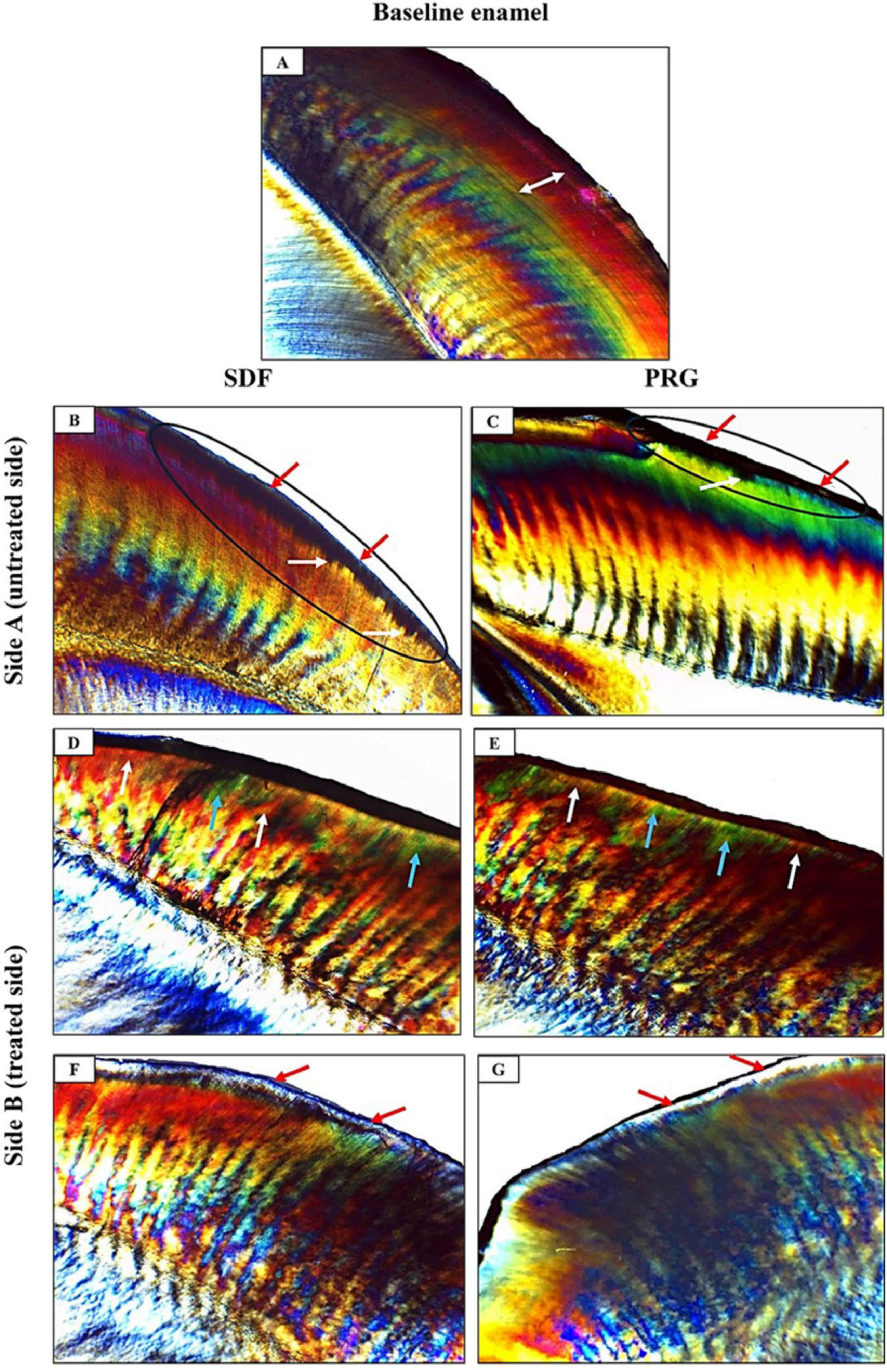
Polarized light photomicrographs of an L.S ground section of Baseline normal enamel, side A (untreated side) of SDF and PRG groups, and side B (treated side) of SDF and PRG groups. **A** **Normal enamel** (Baseline) with a prismatic surface enamel layer with substantially normal uniform subsurface enamel (double head arrow), indicating typical enamel mineralization and birefringence. **B** **SDF-A** is showing surface demineralization and exhibiting a demineralized enamel band (circled area) with a positive birefringence extending under an intact surface layer (red arrows). Notice the penetration of demineralization into deeper enamel (white arrows). **C** **PRG-A** showing surface demineralization with a positive birefringent demineralized enamel band (circled area) under a surface layer that appears intact (red arrows) and extension of demineralization into deeper enamel (white arrow). **D** **SDF-B** shows widely distributed areas of remineralized enamel (white arrows) together with small, demineralized areas (blue arrows). **E** **PRG-B** showing alternative areas of remineralized enamel (white arrows) together with small, demineralized areas (blue arrows). **F** **SDF-B** showing an apparent remineralized surface enamel layer (red arrows). **G** **PRG-B** shows the elimination of demineralization with the appearance of a surface remineralized layer (red arrows).

PLM for Baseline normal enamel showed a standard prismatic surface enamel layer with almost typical homogenous subsurface enamel reflecting normal mineralization and birefringence of enamel (Fig. 5A). However, side A of SDF (untreated side) showed surface demineralization with a positive birefringent demineralized enamel band extending beneath an intact surface layer (Fig. 5B). Similarly, side A of PRG depicted surface demineralization with a positive birefringent demineralized enamel band beneath an intact surface layer and an apparent extension of demineralization into deeper enamel (Fig. 5C). The treated side of SDF showed widely distributed areas of remineralized enamel, small, demineralized regions, and an evident remineralized surface enamel layer (Fig. 5D, F). Furthermore, the treated side of PRG showed alternative areas of remineralized enamel together with small, demineralized areas and elimination of demineralization with the appearance of a surface remineralized layer **(**Fig. 5E, G).

## Discussion

A chronic issue with orthodontic treatment is the demineralization of the enamel around the brackets, which is frequently attributed to poor oral hygiene [31]. WSLs caused by orthodontic treatment pose a significant difficulty in reaching the desired aesthetic outcome [32]. Numerous methods were tried to protect dental enamel from cariogenic challenges, primarily the development of WSL because the enamel is not regenerative [27, 33–35].

Our study compared the efficacy of utilizing a PRG barrier coat versus a silver diamine fluoride varnish treatment before bonding orthodontic brackets. Due to their availability, the method shown insensitivity, and the well-known potential to arrest caries, these two materials were chosen to be investigated [19, 36]. Here, our suggestion was to assess their impact if used during bracket bonding, which, if effective, would be feasible and not demand patient cooperation. Furthermore, being protected from the oral environment by the overlying orthodontic bonding agent and brackets would benefit our proposed technique. Tested materials were applied after enamel etching because it was suggested that the etched enamel surface has higher surface energy and shows increased absorption of applied materials [37]. For the PRG-barrier coat group, the coat was applied and cured before primer application to ensure penetration of the material’s active components to the tooth structure, keeping in mind that an oxygen-inhibited layer which forms at the surface of resins during air curing, may enhance interfacial bonding between the two resins; PRG-coat and orthodontic primers, based on molecular interaction principles [38].

The split-tooth model was utilized in this study to minimize variability between the treated and control groups since each tooth serves as its own control [27]. Eliminating any potential bias between different teeth can lead to more precise estimates of treatment effects. Baseline measurements were also performed to determine whether the suggested treatment can maintain the parameters of typical healthy enamel.

Teeth were preserved in artificial saliva to imitate the clinical setting because saliva is a critical element in the demineralization-remineralization cycle [39]. Additionally, cementing brackets were included in our research plan, followed by a simulated cariogenic challenge employing a combination of demineralizing and remineralizing solutions during the pH cycle to assess the anti-cariogenic capabilities during the study [29].

Regarding the analysis at the ultra-structural level, EDX is a valuable micro-analytical technique used in many types of research in conjunction with SEM to measure the number of enamel minerals besides its structural analysis [40]. This was augmented by the Vickers microhardness test, a typical test used to investigate how demineralization or remineralization phases affect the tissues of the teeth [41]. Also, PLM has often been used to explore the demineralization and remineralization of hard tissues [42, 43]. The light is positively diffracted when passing through organic regions (demineralized) and negatively diffracted when passing through inorganic regions (mineralized or remineralized) [44].

Because appropriate retention of orthodontic brackets throughout treatment is essential to success, shear bond strength was measured in both the treated and control groups [45]. Bond strength was essential because the enamel surface was prepared before bracket bonding.

The first null hypothesis was rejected because, after analyzing data from the current study on Ca/P ratios, treated sides from both groups exhibited statistically significantly more significant Ca/P ratios than non-treated sides after pH cycling. This indicates a considerably lower rate of enamel surface decalcification and higher resistance to acid attack [27]. Statistically, the treated side of the SDF group has Ca/P ratios that are not significantly different from baseline ratios. Deery et al. [46] previously proposed that the precipitation of silver salts upon application on hard tooth structures has a blocking effect by forming insoluble silver apatite, thus decreasing the permeability of the tooth surface [46]. For the PRG Barrier Coat treated group, previous studies have concluded that the S-PRG filler has a triple action: antimicrobial, acid neutralization, and mineral deposition, which occurs through increasing the mineral uptake during remineralization phases [23, 47]. This process is mainly assisted by fluoride, silicate, and strontium, which can interact with hydroxyapatite, forming strontium-apatite with improved acid resistance [48, 49].

**SEM** and **PLM results** showed a protective effect of **SDF** through enamel remineralization along the whole enamel surface with many large calcium crystals combined with small porosities between the calcium crystallites. This finding was agreed upon by Rossi et al. [50] who reported a strong surface enamel remineralization after SDF application. This could be attributed to the presence of calcium fluoride and silver phosphate [51]. Moreover, the hydroxyl ions of hydroxyapatite are combined with fluoride ions to form fluorapatite. Silver phosphate also helps in the formation of fluorapatite [52]. In addition, the PRG-coat group showed nearly the same histological results. This was agreed by Iijima et al. [53].

The remineralization potential of the two varnishes was evaluated by measuring enamel hardness using a microindenter. The results demonstrated that baseline values of VHN were higher for both groups than for treated sides, which were significantly higher than for non-treated sides, thereby rejecting the first null hypothesis once more. This is consistent with Ca/P measurements demonstrating mineral deposition in both groups. However, the reduced hardness values on the treated sides could be attributed to the fact that frequent contact with the demineralizing solution during pH cycling has a greater influence on the external surface than on deeper regions, as was reported in a previous study [13]. Another issue relevant to our investigation is that the treated groups were additionally challenged by the acid etching that occurred before bonding [54]. Despite these challenges, we must acknowledge that enamel hardness was restored by around 80% in both treatment groups.

The findings of our study are consistent with earlier research indicating that coatings containing S-PRG result in enhanced Knoop microhardness values of bovine enamel when compared to untreated enamel [13, 25]. In a related study by Reis et al. [10], the effectiveness of silver diamine fluoride (30%) was compared to that of a bioactive giomer light-curing varnish in terms of their ability to halt non-retentive caries, assessed through Knoop microhardness of dentin. Their results demonstrated that specimens treated with the giomer exhibited greater microhardness than the untreated samples, while showing comparable values to those in the SDF group. Conversely, the SDF group did not present any statistically significant differences when compared to the control group.

When comparing the effect of the two materials using the mean difference between their treated sides and baseline, there was no statistical difference between them regarding Ca/P ratios and hardness. This is consistent with Ca/P measurements demonstrating mineral deposition in both groups.

The second null hypothesis was accepted as SBS did not differ significantly between the groups. The two applied varnishes showed slightly higher SBS compared to the control group. The high SBS values obtained from the SDF group may be due to its reaction with enamel crystals, which produces calcium fluoride and silver phosphate, predisposing to surface alteration and high bonding [55, 56]. Regarding the PRG-coat group, some factors may explain how SBS was adequate; it can adhere to the tooth surface without etching and its ability to be a 15-µm film [24]. Besides, the mode of application in our study took advantage of the presence of oxygen inhibited layer for better bonding with the orthodontic adhesive [37]. As a noteworthy point, all groups exhibited stronger mean shear bond strengths than those required for successful orthodontic bonding. Reynolds [57] found that clinically acceptable bond strengths ranged from 5.9 to 7.8 MPa.

Our findings agree with a previous study which concluded that using SDF did not have a negative effect on the μ-SBS of composite resin when it was used on intact or demineralized enamel [56].

The limitations of this in vitro investigation were the inability to imitate the oral environment in terms of the salivary biofilm and oral bacteria, varied salivary components coupled with different individual eating habits, and dental hygiene practices. Experimental studies are typically conducted over a relatively short period, which may limit the ability to assess the long-term effects of tested materials. In clinical terms, these materials could potentially play a significant role in caries prevention. However, the existing limitations prompt the authors to recommend conducting further long-term clinical trials, especially in cases characterized by a high caries index or poor oral hygiene, to investigate the preventive effectiveness of these materials in such environments.

## Conclusions

According to the results of this study, we can conclude that applying either SDF varnish or PRG-barrier coat before bonding orthodontic brackets could effectively prevent the development of WSL and achieve surface enamel protection. In addition, the two applied varnishes showed slightly higher shear bond strength of orthodontic brackets compared to the control group, with the SDF slightly higher than PRG. These findings indicate that incorporating these protective agents in orthodontic practice may enhance enamel preservation without compromising bracket adhesion. However, further clinical studies are necessary to confirm these results in vivo.

## Data Availability

The datasets utilized and/or analyzed during the present study are accessible from the corresponding author upon reasonable request.
